# How Shall the Physician Disinfect His Hands?

**Published:** 1885-09

**Authors:** 


### How shall the Physician Disinfect his Hands?

Dr. Forster, of Amsterdam, Holland, after a careful series of experiments in the Hygienic Institute at Amsterdam, concludes that a solution of carbolic acid is unreliable; that a diluted solution of corrosive sublimate, one to two thousand, is absolutely reliable. Therefore, he urges all physicians to so disinfect their hands, before leaving any case from which they may convey disease, be it any of the exanthemata or a surgical case.

